# Morphological and niche divergence of pinyon pines

**DOI:** 10.1002/ece3.1994

**Published:** 2016-03-23

**Authors:** Alejandra Ortiz‐Medrano, Daniel Patrick Scantlebury, Alejandra Vázquez‐Lobo, Alicia Mastretta‐Yanes, Daniel Piñero

**Affiliations:** ^1^ Departamento de EcologíaInstituto de EcologíaUniversidad Nacional Autónoma de MéxicoAP 70‐275Mexico CityMéxico; ^2^ ResonateRestonVirginiaUSA; ^3^ Centro de Investigación en Biodiversidad y ConservaciónUniversidad Autónoma del Estado de MorelosCuernavacaMéxico; ^4^ CONACYT Research Fellow assigned to CONABIOCoordinación de Análisis de Riesgo y BioseguridadDirección General de Análisis de PrioridadesComisión Nacional para el conocimiento y uso de la BiodiversidadMexico CityMexico; ^5^ Present address: Universidad del Medio AmbienteCamino al Castellano 4Valle de BravoMéxico

**Keywords:** Adaptation, ecological niche modeling, niche conservatism, niche divergence, phylogenetic adaptive methods, *Pinus* subsection *Cembroides*

## Abstract

The environmental variables that define a species ecological niche should be associated with the evolutionary patterns present in the adaptations that resulted from living in these conditions. Thus, when comparing across species, we can expect to find an association between phylogenetically independent phenotypic characters and ecological niche evolution. Few studies have evaluated how organismal phenotypes might mirror patterns of niche evolution if these phenotypes reflect adaptations. Doing so could contribute on the understanding of the origin and maintenance of phenotypic diversity observed in nature. Here, we show the pattern of niche evolution of the pinyon pine lineage (*Pinus* subsection *Cembroides*); then, we suggest morphological adaptations possibly related to niche divergence, and finally, we test for correlation between ecological niche and morphology. We demonstrate that niche divergence is the general pattern within the clade and that it is positively correlated with adaptation.

## Introduction

Phenotypic features of organisms that enable them to survive in diverse environments have long fascinated evolutionary biologists who have sought to identify characters shaped by natural selection that increase the individual fitness in specific environments. These adaptations, being the result of selective pressures over phenotypes, often come in the form of morphological changes that reflect the habitat or environment in which species thrive. Hence, the ecological niche understood as environmental variables and conditions that define ecological properties of species (Grinellian niche sensu Soberón 2007; Peterson 2011) should reflect the evolutionary pattern seen in the adaptations that have resulted from these same variables. As a consequence, when comparing across species, we can expect to find an association between phylogenetically independent phenotype characters and ecological niche evolution.

There are two main hypotheses regarding evolutionary processes associated with the ecological niche: niche conservatism and niche divergence (Peterson 2011). The first proposes that niche conservatism happens when two species retain ancestral ecological characteristics, promoting their geographic divergence, which in turn could induce phylogenetic diversification (Wiens and Graham 2005). Under this scenario, speciation can happen when allopatric lineages with conserved niches are unable to tolerate climatic conditions between their ranges (Kozak and Wiens 2006). The second states that lineages occupying different environmental conditions will adapt each one to its environment leading to different adaptations and phylogenetic divergence.

Because of the role of environment on shaping species traits through adaptation, niche conservatism and niche divergence should be closely tight to morphological divergence. To date, few studies have evaluated how organismal phenotypes might mirror patterns of niche evolution as would be expected whether these phenotypes were reflecting adaptations (Cicero and Koo 2012; Fontanella et al. 2012; Forrestel et al. 2015). Doing so would contribute to the understanding of the origin and maintenance of phenotypic diversity observed in nature, particularly on the importance of natural selection as a driving force both for niche evolution and for morphological adaptations.

Here, we first describe the niche evolution pattern within the North American pinyon pines lineage (*Pinus* subsection *Cembroides*); then, based on a comparative method approach, we suggest morphological adaptations possibly related to niche divergence, and finally, we test for niche and morphological correlation. We demonstrate that niche divergence is the general pattern of the clade and that it is positively correlated with character adaptation.

We use pinyon pines as our study system for four main reasons. First, they are a diverse (11 species, 10% of the *Pinus* species, Price et al. 1998) monophyletic group whose recent divergence (9.5–16 Mya; Gernandt et al. 2008) is likely associated with the establishment of arid and semiarid environments of North America during Late Miocene and Pliocene (Wilson and Pitts 2010). Second, their distributions are mainly at the boundaries of arid regions and commonly their populations are scarce, isolated, and often restricted to a very specific habitats (Perry et al. 1998). Third, when compared with other pine groups, pinyon pines display an outstanding morphological diversity, suggesting that adaptation could play an important role in the diversification of this group, and indeed, some morphological adaptations have been suggested based on observations and correlations with humidity clines, but no work has been carried out using comparative phylogenetic methods (Malusa 1992; Poulos and Berlyn 2007, Cole et al. 2008). Fourth, these species occur in the Mexican highlands, a biodiversity hotspot hypothesized to promote divergence due to its high habitat heterogeneity (Halffter 1987; McCormack et al. 2008; Morrone 2010; Ruiz‐Sanchez and Specht 2013). Therefore, the pinyon pines are a compelling system to explore the role of adaptation within the framework of phylogenetic relationships and to test hypotheses of morphological and niche evolution.

## Methods

### Phylogenetic analyses and model fitting

We built a phylogeny of subsection *Cembroides* using nearly complete chloroplast genomes sequences (ca. 116,848 bp, Parks et al. 2012) with 11 pinyon pine species, with *Pinus nelsonii* and *Pinus ayacahuite* plastomes as outgroups. To do so, we aligned their sequences with MAFFT v. 6 (Katoh et al. 2002) with the default settings and we conducted four independent searches in RaxML v8 (Stamatakis 2014) with the quick maximum likelihood algorithm. We obtained the same topology for the four best trees, so we used the tree with the highest likelihood to serve as the starting tree for Bayesian searches. This tree was transformed to be ultrametric using the function *chronopl* of *ape* with *λ* = 1 (Paradis et al. 2004) and rescaled to a length of 100 with the function *rescaleTree* of the *geiger* package (Harmon et al. 2008) of the R statistical software v 3.1.2. (R Development Core Team, 2011). Bayesian searches were carried out in BEAST v 1.7 (Drummond et al. 2012) with 20 million of MCMC generations, sampling every 10,000 generations. We assumed a GTR model of evolution following a gamma distribution and a lognormal relaxed clock for these searches.

We tested stability of topologies and parameters with Tracer 1.5 (Drummond et al. 2012) and AWTY (Wilgenbusch et al. 2004). These analyses revealed that the first million MCMC generations could be discarded as preburn in values and that all searches converged on a similar set of topologies and parameter estimates. Finally, we computed the maximum clade credibility tree with node heights set to the mean of the posterior distribution as a summary of topological uncertainty.

### Characterization of ecological data

**Figure 1:**
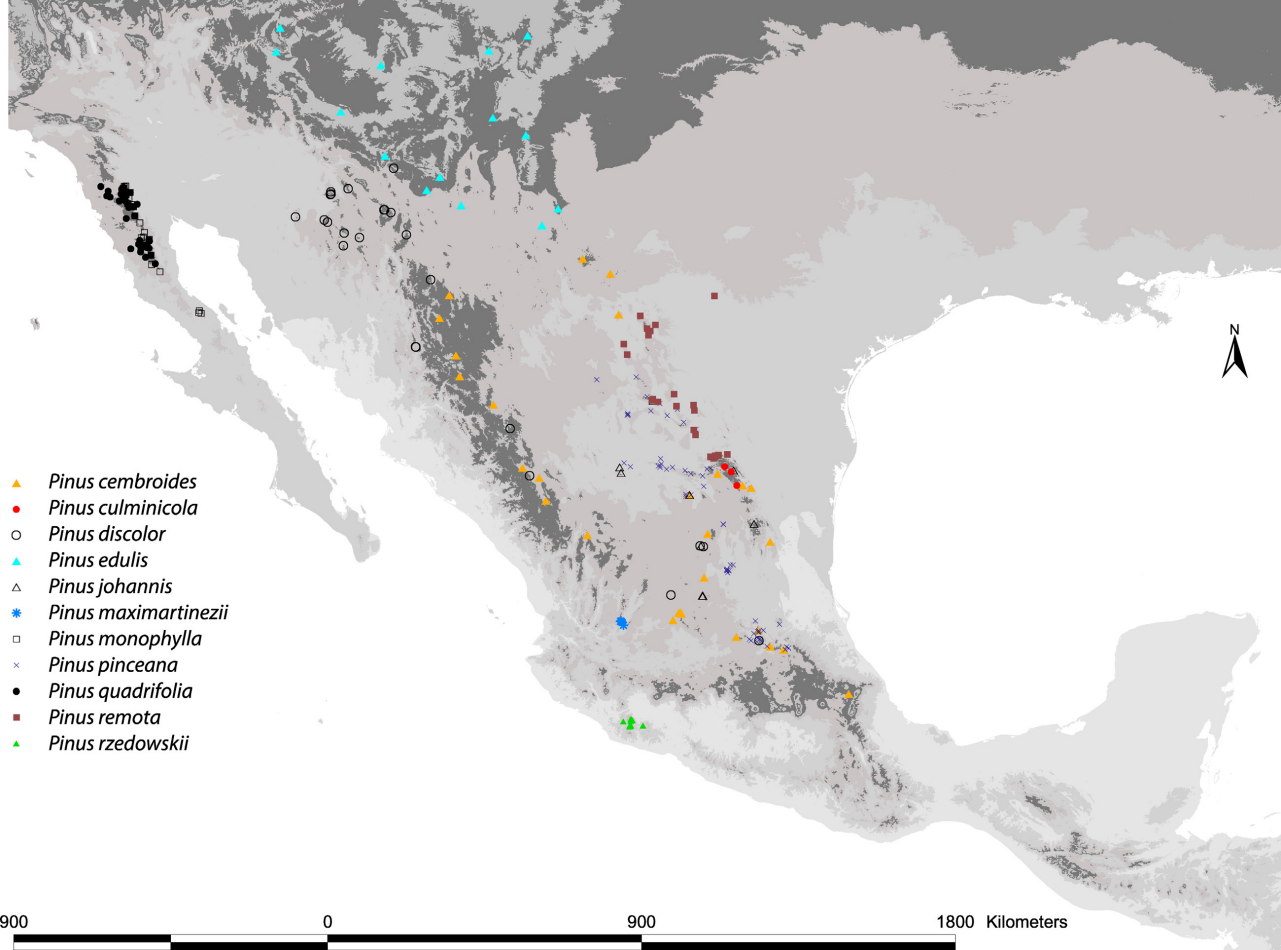
Geographic localities used.

We searched for localities of the 11 pinyon pines species recorded in the literature (Cuenca 2001; Flores‐Rentería et al. 2013), the Red Mundial de Información sobre Biodiversidad (INEGI‐CONABIO‐INE, 2008) and personal field collections by Patricia Delgado. We restricted the database to records with coordinates reporting seconds and certainty in the species identification, either by genetic confirmation from previous analyses (Cuenca et al. 2003; Flores‐Rentería et al. 2013) or by recent expert identification by David Gernandt. A total of 261 localities were used ranging from three localities (*P. culminicola*) to 57 localities (*P. pinceana*), representative of the known localities of the species (see Fig. 1 and Data S1). Environmental information for each record was extracted from 19 bioclimatic layers with a 30 arcsecond resolution (Bioclim; Hijmans et al. 2005). To avoid redundancy in environmental information, we performed pairwise correlations among all variables; then, from groups or pairs highly correlated (*r* ^2^ > 0.75), we chose the variables that best explain the distribution of pines, based on previous ecophysiological and distribution studies (Poulos and Berlyn 2007; Poulos et al. 2007; Poulos 2009). We restricted our analyses to ten variables: maximum temperature of the warmest month, temperature seasonality, mean temperature of the driest quarter, mean temperature of the coldest quarter, annual precipitation, precipitation of the driest month, precipitation seasonality, precipitation of the driest quarter, precipitation of the warmest quarter, and mean diurnal range.

### Morphological data

We analyzed ten morphological characters: shell thickness, shell length, shell width, needle length, needle width, number of needles per fascicle, tree height, cone scale thickness, cone width, and cone length. Most of these characters have been previously discussed to potentially present climate‐related adaptations in some pinyon pine species (Malusa 1992). Most measurements came from a variety of sources (Malusa 1992; Nobis et al. 2012; Flores‐Rentería et al. 2013), and the rest from our own efforts on *P. rzedowskii* cone and seed measurements.

### Phylogenetic model fitting

Phylogenetic comparative methods hinge on the assumption that covariances between species are phylogenetically nonindependent (Felsenstein 1985). However, these methods are not appropriate when the observed character data do not show phylogenetic dependence (Losos 2008; Revell 2010). Thus, we evaluated the fit of our data to nine models of character evolution prior to performing comparative methods: eight models incorporate some degree of phylogenetic dependence, and in one, character data is phylogenetically independent and drawn from a random distribution (i.e. “white noise” model). We performed model fitting with the *fitContinuous* function from the *geiger* package (Harmon et al. 2008) in R v 3.1.2.

We used the maximum likelihood estimate of Pagel's lambda (Pagel 1999) to transform the variance–covariance matrix. Lambda estimates were small for all trees (<0.01, data not shown). We performed lambda estimation and model fitting on the MCC tree as well as 99 random trees from the posterior distribution to account for phylogenetic uncertainty.

### Pattern of ecological niche evolution

We compared species ecological niche models (ENM) to distinguish between niche conservatism and niche divergence between species. Niche models were constructed using Maxent v. 3.3.3e with default parameters (Phillips et al. 2010), using the ten bioclimatic variables mentioned above. We used the background test of ENMTools v 1.3 with 100 replicates (Warren et al. 2010) to compare niches between species. This approach tests whether two niches are more different or similar than expected by chance, given the available environment for both species. This available environment is called “background,” and the result of the test is very sensitive to the choice of background (Warren et al. 2010).

We defined the background according to the ecoregions described for Mexico in *Ecorregiones Terrestres de México* (INEGI‐CONABIO‐INE, 2008). For each species, we chose as background the regions in which there was at least one occurrence point. For *P. edulis*, which mainly occurs outside of Mexico, we constructed a buffer of 100 km surrounding each locality point and used this as background. We used Warren's I (Warren et al. 2010) as metric for niche divergence, in order to use Hellinger distances as a proxy for niche divergence in the Mantel test detailed below.

### Morphological adaptations

To test whether pine morphology covaries with climate as expected from our hypotheses about niche evolution, we used two‐block partial least squares (2B‐PLS), a type of Eigen analysis that is useful for exploring patterns of covariation between two sets of variables (Rohlf and Corti 2000). 2B‐PLS takes two sets of observations (in our case, the mean of ten bioclimatic variables and ten morphological variables) and constructs linear combinations of variables within each matrix such that new variables account for as much of the original covariance between the original variables as possible (Rohlf and Corti 2000). This analysis preserves the geometry of the original data, enabling examination of multidimensional patterns. We used the *Rv* coefficient (Robert and Escoufier 1976) to summarize the amount of covariance in each dataset that is accounted for by the other dataset.

We performed the 2B‐PLS analysis on the correlation matrix from the environmental and morphological datasets. We assessed significance of all 2B‐PLS summary statistics via permutation of the rows of either dataset; significant observed values are in the 95 percentile of simulated values. We performed all 2B‐PLS analyses in R with our own code (Data S2).

### Niche evolution and morphological adaptations

We tested whether niche divergence between species is associated with morphological divergence using a Mantel test with 999 permutations using the *ade4* package (Chessel et al. 2004) of R. As a measure of niche distance, and to be able to compare the results of niche evolution with morphological divergence, we used Hellinger distances between niche models as proposed by Warren et al. (2010). We used Hellinger's distance and not the *I* statistic because Mantel tests operate on distances, whereas the *I* statistic is a summary of how close two niche models are to the maximum possible Hellinger distance. The two statistics are directly related (*I *=* *1 − (*H* ^2^)/2), but they deal with different aspects of divergence. Hellinger distances address the absolute differences between probability distributions, whereas the *I* statistic addresses the distance that two distributions are from the maximum possible divergence. As outputs from our niche model analyses are logistic suitability scores, we first standardized scores for each species to sum to 1 and then computed the Hellinger distance.

To estimate morphological distances, we used standard Euclidean distances between species in morphospace using our own R code (Data S3).

## Results

### Phylogenetic analysis and model fitting

**Figure 2:**
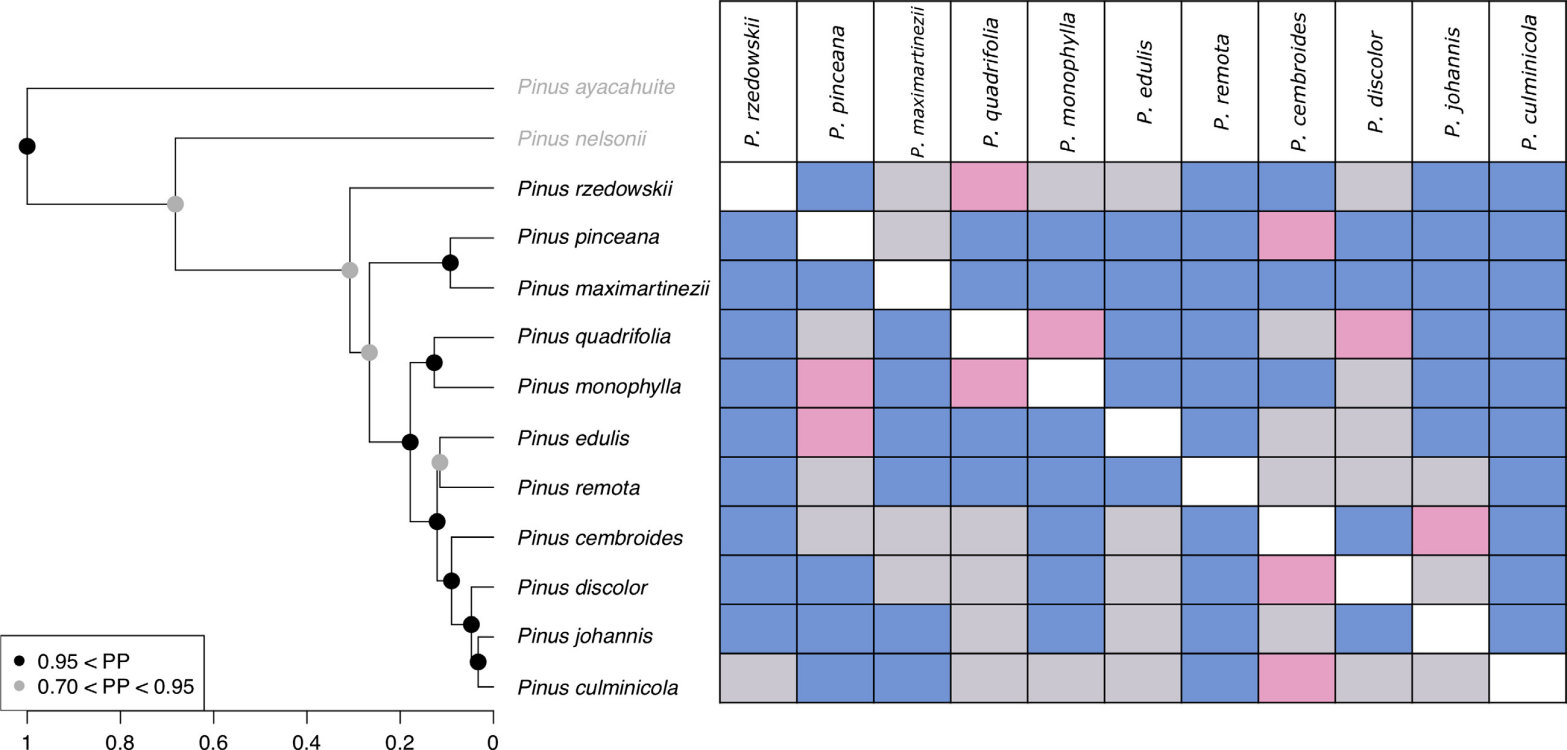
Maximum clade credibility tree and niche evolution pattern. Niche divergence dominates the niche evolution pattern of the clade. Circles on the nodes of the tree indicate posterior probability support values. Blue cells indicate divergence, pink conservatism, and gray nonsignificant.

The topology of the tree is shown in Figure 2. All nodes are supported by a posterior probability of at least 0.70, and most (seven of 11) are at least 0.95. The topology recovered is consistent with previous phylogenetic inferences (Gernandt et al. 2003, 2005; Parks et al. 2012), but may have inconsistencies due to introgression as has been described for this and other pine lineages (e.g., Gernandt et al. 2003; Delgado et al. 2007; Liston et al. 2007; Hernández‐León et al. 2013). Further works focused on phylogenetic inferences for this group should consider inclusion of nuclear and/or mitochondrial markers and individuals from allopatric populations to elucidate a more complex history of this lineage (Gernandt 2003).

Most of the posterior trees for both datasets favoured the white noise or nonphylogenetic model, where there is no covariance structure among species (Butler and King 2004; data not shown). As these results do not show evidence for phylogenetic dependence in our data, we tested for adaptation without assuming the influence of phylogeny on covariance patterns among pine species.

### Pattern of ecological niche evolution

All niche models showed area under the curve (AUC) values higher than 0.9. For the background test that measures paired niche similarity, in most cases, at least one of the reciprocal comparisons was significant. Overall, our results indicate a pattern of niche divergence in the pinyon pines (Fig. 2). However, paired comparisons between *P. quadrifolia*–*P. cembroides*,* P. edulis–P. cembroides*,* P. edulis–P. discolor* resulted nonsignificant in both directions. In some cases, reciprocal comparisons showed significant but opposite results (i.e., niche divergence in one way, niche conservatism in the other, Table 1), suggesting that conserved niches are nested within the divergent ones, that is, the niche of the species that shows divergence has all the conditions observed in the conserved one, but not the other way around. Only one pair shows conservatism in both ways, *P. monophylla–P. quadrifolia* (Fig. 2).

**Table 1:** Number of conserved, divergent, and nonsignificant paired comparison per species

Species	Conserved	Divergent	NS
*P. culminicola*	1	13	6
*P. johannis*	1	13	6
*P. discolor*	2	9	9
*P. cembroides*	4	8	8
*P. remota*	0	16	4
*P. edulis*	1	12	7
*P. monophylla*	3	14	3
*P. quadrifolia*	4	10	6
*P. maximartinezii*	0	16	4
*P. pinceana*	3	13	4
*P. rzedowskii*	1	14	5

### Morphological adaptations and niche divergence

**Figure 3:**
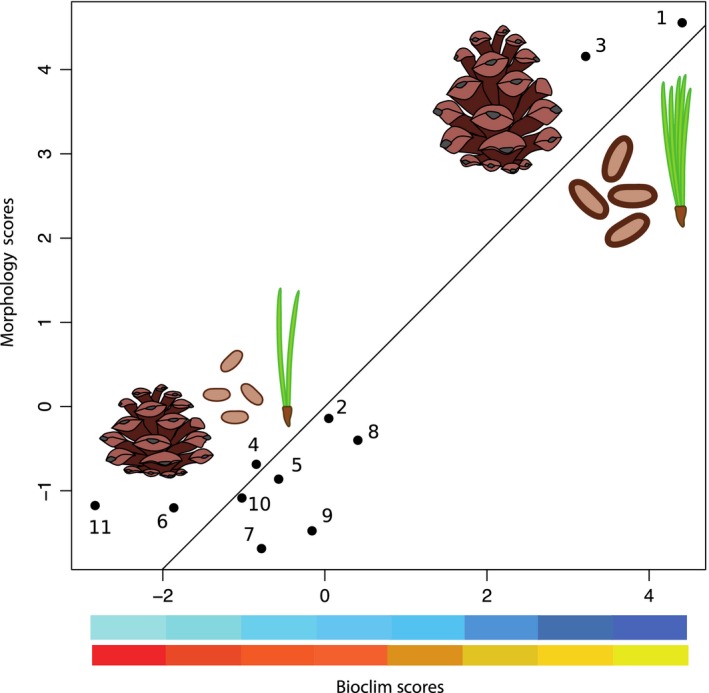
Covariation between morphological and bioclimatic matrices suggests adaptation. A colder and wetter environment covaries with an increase in needle number and cone and seed dimensions. Numbers indicate 1 *Pinus rzedowskii*, 2 *P. pinceana*, 3 *P. maximartinezii*, 4 *P. quadrifolia*, 5 *P. monophylla*, 6 *P. edulis*, 7 *P. remota*, 8 *P. cembroides*, 9 *P. discolor*, 10 *P. johanis*, and 11 *P. culminicola*.

The 2B‐PLS analysis revealed a strong association between morphology and climate, which is consistent with our hypothesis regarding adaptation (Fig. 3). Overall, 64.07% of the covariance in morphology is explained by climate conditions (*Rv* = 0.6407, *P *=* *0.009). The first latent dimension summarizes 84.02% of the total observed covariance (*P *<* *0.001), and the correlation between latent variables along this axis was strong and robust to permutation tests (*r *=* *0.925, *P *=* *0.0193, Table 2). Given that *Pinus maximartinezii* and *Pinus rzedowskii* seemed to be driving the results (Fig. 3), we conducted a second 2B‐PLS analysis removing these species. In this analysis, 42.45% of the covariance in morphology is explained by climatic conditions (*Rv* = 0.4245, *P *=* *0.03), and the first two latent dimensions summarized 91.83% of the total observed covariance (*P *<* *0.0001).

**Table 2:** Covariation between morphological and bioclimatic matrices

Matrix	Variable	Dimensions	
		1	2
F_1_	Precipitation seasonality	0.4634	−0.1116
	Precipitation of warmest quarter	0.4274	0.2310
	Annual precipitation	0.4226	0.3331
	Mean temperature of coldest quarter	0.3522	−0.0788
	Temperature seasonality	−0.3259	−0.2192
	Mean temperature of driest quarter	0.3055	−0.3355
	Precipitation of driest quarter	−0.2625	0.3002
	Precipitation of driest month	−0.1752	0.3013
	Mean diurnal range	−0.0239	−0.4573
	Max temperature of warmest month	0.0160	−0.5162
F_2_	Shell thickness	0.4340	0.2651
	Cone length	0.4189	−0.2250
	Cone width	0.3880	−0.3718
	Shell width	0.3534	0.4030
	Tree height	0.3226	0.0726
	Shell length	0.3102	−0.1981
	Cone scale thickness	0.2975	−0.4377
	Needles	0.2569	0.5842
	Needle width	−0.0733	0.0014
	Needle length	0.0220	−0.0318
Singular value		4.2608	1.5296
Proportion total covariance explained		0.8403	0.1083
Correlation		0.9206	0.7705

**Figure 4:**
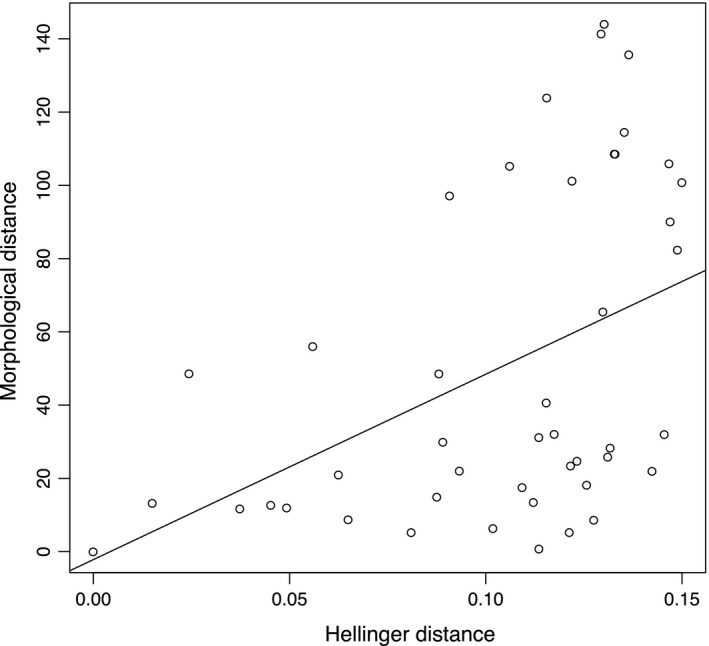
Mantel test between Hellinger (log) and morphological distances shows a positive correlation.

Finally, niche and morphological divergence were positively correlated (Mantel test *r* ^2^ = 0.6, *P *=* *0.001, Fig 4).

## Discussion

### Phylogenetic analysis and model fitting

For ecological and morphological datasets, the majority of posterior trees favoured the white noise model. We interpret that these results indicate that our data are not displaying evidence of phylogenetic dependence, so we tested for adaptation without assuming the influence of phylogeny on covariance patterns among pine species (Butler and King 2004).

### Pattern of ecological niche evolution

Overall, our results indicate a pattern of niche divergence in the pinyon pines. Although in some cases the paired niche similarity test found significant results for both niche divergence and niche conservatism, most of the comparisons (70 of 110, Fig. 2, Table 1) support the notion that niche divergence is a general trend for this clade. Niche divergence patterns have been detected in other groups (beetles, Sánchez‐Fernández et al. 2011; rodents, Kalkvik et al. 2012; snakes, Wooten and Gibbs 2012), and although niche conservatism seems to be the norm in recently diverged lineages, this conservatism breaks down with time (Peterson 2011). Pinyon pines, although of recent divergence within the conifers (between 9.5 and 16 million of years of divergence, Gernandt et al. 2011) is considered separate species (Gernandt et al. 2003, 2011; Flores‐Rentería et al. 2013), so niche divergence could be a consequence of speciation. Nevertheless, as in any niche modeling research, the pattern could be the result of the methodological artifacts discussed below.

The divergence pattern detected could be a methodological artifact arising mostly as a result of the environmental data set used, because as a rule of thumb, the more environmental dimensions, the more likely a divergence pattern will arise, given that there are more variables in which species can diverge. As Wiens and Graham (2005) put it “species will always inhabit environments that bear some similarity to those of their close relatives (i.e., few tropical rainforest species have a sister species in undersea vents). Thus, to some extent, niches will always be conserved. Yet, few sister species may share identical niches; so niches may never be perfectly conserved either.” Therefore, whether or not niches are conserved depends on how much similarity is worthy of being considered conservatism. We cannot dismiss that some degree of methodological artifact is biasing our results. However, to account for this, we reduced our environmental variables to avoid correlation and to keep the more relevant ones based on ecophysiological and distribution studies (Poulos and Berlyn 2007).

Another common source of artifacts is the methods used to measure the niche conservatism (Peterson 2011). The background test we used here was developed explicitly to be a counterweight of very astringent tests for niche conservatism, such as the identity test (Warren et al. 2010). Such tests could produce false positives because are prone to interpret their results as niche divergence, given that the hypothesis to reject is that the niches are identical, which is rarely the case, so the hypothesis of niche identity is seldom rejected. The background test, on the other hand, has a very clear working hypothesis that is less likely to bias results toward niche divergence, because niches can be conserved but not identical (Warren et al. 2010; Peterson 2011). This, along with our environmental variable reduction, reduces the methodological artifact chances of the results.

### Morphological adaptations

The 2B‐PLS analysis revealed a strong association between morphology and climate. Furthermore, climate accounted for 64.07% of the covariance in morphological variables when including all species, and 42.45% when excluding *P. maximartinezii* and *P. rzedowskii*. Given that the first latent dimension summarizes a high fraction of the total observed covariance (84.02% for all species and 91.83% when excluding *P. maximartinezii* and *P. rzedowskii*), we restrict our discussion below to this first dimension.

The first dimension has an environmental gradient of seasonal and increasing moisture, along with a declining and stable temperature over the year. Morphologically, this dimension shows an increase in seed shell or coat and cone dimensions, tree height, and a decreasing needle number per fascicle (Fig. 3). A cold and moist gradient defines subsection *Cembroides* distribution, with humidity being a primary factor determining it. In regions where these trees thrive, elevation is associated to higher humidity and lower temperatures. There are also differences in seasonality between lower and higher elevations: Lower altitudes tend to have hot and dry climate most year round, while the higher ones are more variable, given that their summers are wet and their winters have intermittent freezing peaks (Poulos and Berlyn 2007). In our results, this seasonality in precipitation and temperature shows the same trend, and it is in line with some previous ideas regarding morphological adaptations in pinyon pines (Malusa 1992; Richardson et al. 1998).

Among the morphological differences studied here, needle number, cone and seed dimensions have been previously identified as potential adaptations: a decrease in needle number and cone and seed dimension is related to water stress (Malusa 1992; Richardson et al. 1998; Cole et al. 2008) because in arid conditions, less transpiration surface gives an advantage as an adaptation to drought. Similarly, although dispersal may be playing a role in seed an cone dimensions as adaptations (thicker seed shells attracting strong billed corvids, while thinner seed shells appear to attract rodents, Malusa 1992; Vander Wall 1997; Chambers et al. 1999), the covariation of these structures with colder and wetter climates in our results seems to be related to another factor, possibly nutrient availability or other environmental resource (Richardson et al. 1998), which could also explain the tree height increase as a strong variable in the covariation pattern.

Interestingly, when *P. maximartinezii* and *P. rzedoskii* are removed from the analysis, covariance between morphology and climate declines (from 64.07% to 42.45%). These two species represent, along with *P. pinceana*, the earliest diverging lineages in the phylogeny and inhabit the wettest locations of the entire subsection. It has been suggested (Malusa 1992; Farjon 1996) that the evolution of the pinyon pines has been driven by droughts and intense solar radiation that have selected short and rigid needles, as well as a reduction on needles per fascicle. This suggests that the ancestral characters are probably the ones related to wet and cold environments, and thus explaining why *P. rzedowskii P*. *pinceana* and *P. maximartinezii* are the most divergent species in the morphology and climate covariance (Fig. 3); we believe that the rest of the subsection has diverged from the wet and cold ancestral conditions, and have had less time to diverge between them.

The niche evolution pattern (Fig. 2) shows a somewhat similar pattern. Niche divergence is the most common result in all species, but there are a few cases of niche conservatisms, especially among *P. cembroides*,* P. johannis,* and *P. discolor*. These three species are sympatric in at least some part of their ranges, and there has been considerable debate over their phylogenetic relations and even their identities as species, subspecies or varieties (Perry 1991; Farjon and Styles 1997; Price et al. 1998); along with *P. culminicola*, they are considered a complex group with little morphological and genetic divergence (Flores‐Rentería et al. 2013). Our results show that between some pairs of these species there has been niche conservatism which can be expected given their close phylogenetic relations and shared geographic ranges.

*Pinus rzedowskii* and *P. maximartinezii*, considered relictual species of the subsection (Gernandt et al. 2001), showed more niche divergence than the rest of the species (Fig. 2). These two species also appear as more divergent than the others in the morphology and climate covariation (Fig 3). Taken together, our results show that niche divergence is associated with morphological divergence and that this is stronger in older clades.

*Pinus pinceana* is an exception. This species is also considered relictual (Gernandt et al. 2001), but showed niche conservatism with some species and further in morphology and climate from *P. maximartinezii* and *P. rzedowskii* (Fig. 3). *P. pinceana* has the largest genetic diversity in the clade, and its broad but localized geographic range has led to believe that it had a larger past distribution occupying diverse climates (Figueroa‐Corona 2014). We believe that *P. pinceana* ancestor, with large genetic, morphological and ecological diversity, was able to colonize drier regions.

### Morphological divergence has accompanied niche divergence of pinyon pines

Niche divergence and morphological divergence were positively correlated. This study is one of the few attempts to test a direct correlation between morphological change resulting from adaptation and niche divergence (but see Pérez 2009) and confirms our hypothesis that adaptations have accompanied niche shifts in pinyon pines. Several studies have found an association between niche and morphology, mostly within species. For example, Fontanella et al. (2012) and Cicero and Koo (2012) found a correspondence between morphological and ecological niche model divergence between subspecies. In a similar way, Ribeiro et al. (2014) concluded that niches are a diversifying force in morphological features. However, those studies based their conclusions on coincident patterns of niche and morphology independent tests, not in a direct correlation of both features. Pérez (2009) attempt is more similar to ours, as he also used a group of species to directly test the effect of different niches on morphological adaptations, but using discrete habitat categories as a niche measure.

As in most approaches to test morphological adaptations, phenotypic plasticity could be biasing our results. In *Pinus* subsection *Cembroides,* there is no common garden or genetic test to confirm plasticity, although some observations have been made in this regard. *P. cembroides* displays a large variation in morphology, particularly on needle morphology and fascicles per needle (Richardson et al. 1998). This character shows plasticity in other species (Cole et al. 2008), sometimes even in the same tree, which is important because the variation in needles per fascicle has been associated with different precipitation regimes and it is clearly associated with climate (Malusa 1992; Poulos and Berlyn 2007, Cole et al. 2008).

*Pinus maximartinezii* and *P. rzedowskii* show practically no variation in needles per fascicle. In the species where this character is variable, there is a clear association of its variability with climate, especially with precipitation regimes, as our own results suggest (Cole et al. 2008), and apparently, this variation is larger in the most derived species. This may have been important (or continuing to be) in processes where adaptation plays an important role in speciation (i.e., ecological speciation, Rundle and Nosil 2005). Under ecological speciation scenarios, colonization to fluctuant climates promotes the evolution of plasticity and therefore local adaptations (Lande, 2009, Thibert‐Plante and Hendry, 2010). Our results show that seasonal precipitation is an important covariate of morphology (Table 2), and could have been one of the main drivers of adaptation from the ancestral type (the common ancestor of *P. rzedowskii* and *P. maximartinezii*) to the most derived ones (the most recent divergences).

The scope of our study is not probing ecological speciation, but the fact that niche and morphological divergence have covariated together under a phenotypic plasticity scenario points toward this possibility. To confirm this, more research on reproductive isolation and the genetics of adaptation in this study system would be necessary.

Our results show that in the subsection *Cembroides*, natural selection has been a diversifying force for both niche and morphology and that niche divergence and not conservatism has accompanied morphological change. The relatively recent diversification (Late Miocene; Gernandt et al. 2008) and adaptation of the subsection *Cembroides* could be a result of the complex orography and environmental heterogeneity of Mexico.

Mexico possess large high‐altitude plateaus connecting the main mountain systems, for instance, the Northern and the Central plateaus of the Chihuahuan Desert divide the Sierra Madre Occidental and the Sierra Madre Oriental (Mastretta‐Yanes et al. 2015). During Late Miocene and Pliocene, episodic climatic fluctuations occurred (Metcalfe et al. 2000; Zachos et al. 2001), leading to scenarios of altitudinal migration where species distribution ranges expanded or contracted as result of climate fluctuations, in many cases leading to species having a fragmented distribution across the Mexican highlands (Mastretta‐Yanes et al. 2015). This history of expansions and contractions seems to explain the distribution of genetic variation of tropical and subtropical conifer species (Jaramillo‐Correa et al. 2008; Moreno‐Letelier and Piñero 2009; Gugger et al. 2011; Mastretta‐Yanes et al. 2012), and it is under this scenario that diversification by adaptation and niche divergence could be particularly prone to occur, because these isolated populations would also be subjected to differences in local environmental conditions. In this sense, our study supports the idea that the high habitat heterogeneity of the Mexican highlands promoted niche divergence of pinyon pines, thus contributing to the high species richness of conifers for this region.

### Using ENM to test for morphology‐niche evolutionary associations

Using ENM to test for morphology‐niche evolutionary associations as carried out here has several advantages. First, using bioclimatic data and ENMs broadens the possible niches to be compared, without requiring specific categories of habitat. Second, given the current and pervasive rise of ENMs use in ecological and evolutionary studies, its direct correlation with morphology could be useful to evaluate other features related to niche evolution. Lastly, here we have found that ENM can be used not only to compare divergence between species, but also to test if morphological changes have been related to niche divergence. We used this approach to test for niche and morphological divergence of a clade of pines from the Mexican highlands, but this should be useful for other biodiversity hot spots of the world, for instance for other tropical mountain regions of high heterogeneity.

## Conflict of Interest

None declared.

## Supporting information


**Data S1**. Point localities of *Pinus sp* used on analyses.

**Data S2.** 2B‐PLS R code.

**Data S3.** Morphology‐euclidean distances R code.
