# Supplementary material for: Morphological and niche divergence of pinyon pines
Source: Ecol Evol. 2016 Mar 23;6(9):2886–96. doi: 10.1002/ece3.1994 (PMC4803999; doi:10.1002/ece3.1994)
Supplement: Supplementary file 2 — Data S2. 2B‐PLS R code. [file ECE3-6-2886-s002.rtf]

#non-phylogenetic PPLS## ppls.norm: perform non-phylogenetic partial least-squares analysis (PLS); outputs data listed by Rohlf.## ppls.norm.sim: perform permutations to assess significance of PLS.ppls.norm <- function(X,Y, mode){	rows <- row.names(X)	X <- X[rows,]	Y <- Y[rows,]	data <- as.matrix(cbind(X,Y))	n <- nrow(data)	m <- ncol(data)		if(ncol(X)==1){		xcol <- 1		mX <- 1			}	else{		mX <- ncol(X) # number of X variables		xcol <- 1:mX	}	if(ncol(Y)==1){		mY <- 1		ycol <- mX + mY	}	else{    	mY <- ncol(Y) # number of Y variables    	ycol <- 1:mY + mX	}	xnames <- colnames(X)	ynames <- colnames(Y)		if(mode=="cor"){		R.11 <- cor(X,X)		R.12 <- cor(X,Y)		R.21 <- cor(Y,X)		R.22 <- cor(Y,Y)				means <- apply(X, 2, mean)		std <- sqrt(apply(X, 2, var))		new <- X		for(i in 1:ncol(new)){			new[,i] <- (new[,i]-means[i])/std[i]		}		X <- new				means <- apply(Y, 2, mean)		std <- sqrt(apply(Y, 2, var))		new <- Y		for(i in 1:ncol(new)){			new[,i] <- (new[,i]-means[i])/std[i]		}		Y <- new		data <- cbind(X,Y)					}	else if(mode=="cov"){		R.11 <- cov(X,X)		R.12 <- cov(X,Y)		R.21 <- cov(Y,X)		R.22 <- cov(Y,Y)	}	V <- rbind(cbind(R.11, R.12), cbind(R.21, R.22))		Rv<-sum(diag(R.12%*%R.21))/sqrt(sum(diag(R.11%*%R.11))*sum(diag(R.22%*%R.22)))		SVD <- svd(R.12)	if(mX==1){		F2 <- SVD$u		F1 <- SVD$v		rownames(F1) <- xnames		rownames(F2) <- ynames		colnames(F1) <- paste("dim.", 1:ncol(F1), sep="")		colnames(F2) <- paste("dim.", 1:ncol(F2), sep="")	}	else{		F1 <- SVD$u		F2 <- SVD$v		rownames(F1) <- xnames		rownames(F2) <- ynames		colnames(F1) <- paste("dim.", 1:ncol(F1), sep="")		colnames(F2) <- paste("dim.", 1:ncol(F2), sep="")	}			F.block <- rbind(cbind(F1, matrix(0, nrow=nrow(F1), ncol=ncol(F2))), cbind(matrix(0, nrow=nrow(F2), ncol=ncol(F1)), F2))		S <- diag(t(F.block) %*% V %*% F.block)		s <- matrix(0, length(S), length(S))		diag(s) <- S^-0.5				Rf1f2 <- s %*% t(F.block) %*% V %*% F.block %*% s		colnames(Rf1f2) <- rownames(Rf1f2) <- c(paste("F1.",colnames(F1),sep=""), paste("F2.",colnames(F2), sep=""))				Rscores <- s %*% t(F.block) %*% V		rownames(Rscores) <- colnames(Rf1f2)				ndim <- nrow(Rf1f2)/2				cor.coefs <- diag(Rf1f2[1:ndim,((ndim+1):(ndim*2))])				dC <- matrix(0, nrow=ncol(F.block), ncol=ncol(F.block))		diag(dC) <- 1				cor.resid <- F.block %*% dC %*% Rf1f2 %*% dC %*% t(F.block)				X1 <- data[,	xcol]		Y1 <- data[, ycol]		Xscores <- X1%*%F1		Yscores <- Y1%*%F2		singv <- SVD$d	overall.cov <- sum(singv*singv)/(mX*mY) # mX*mY is the max for correlations	total.cov <- (singv*singv)/sum(singv*singv)	return(list(F1=F1, F2=F2, singv=singv, overall.cov=overall.cov, total.cov=total.cov, Rv=Rv, X.data=X1, Y.data=Y1, Xscores=Xscores, Yscores=Yscores, cor.resid=cor.resid, Rf1f2=Rf1f2, Rscores=Rscores, cor.coefs=cor.coefs))}############ppls.sim.norm <- function(X, Y, nperm, mode){	rows <- rownames(X)	dX <- X[rows,]	dY <- Y[rows,]	rv.sim <- numeric(nperm)	cov.sim <- matrix(NA, nrow=nperm, ncol=min(ncol(X), ncol(Y)))	cor.coefs.sim <- matrix(NA, nrow=nperm, ncol=min(ncol(X), ncol(Y)))	singv.sim <- matrix(NA, nrow=nperm, ncol=min(ncol(X), ncol(Y)))	for(i in 1:nperm){		rand <- sample(1:nrow(X), nrow(X))		X <- dX[rand,]		rownames(X) <- rows		sim <- ppls.norm(X, Y, mode)		rv.sim[i] <- sim$Rv		cov.sim[i,] <- sim$total.cov		cor.coefs.sim[i,] <- sim$cor.coefs		singv.sim[i,] <- sim$singv	}		return(list(rv.sim=rv.sim,cov.sim=cov.sim, cor.coefs.sim=cor.coefs.sim, singv.sim=singv.sim))}
