# Supplementary material for: Morphological and niche divergence of pinyon pines
Source: Ecol Evol. 2016 Mar 23;6(9):2886–96. doi: 10.1002/ece3.1994 (PMC4803999; doi:10.1002/ece3.1994)
Supplement: Supplementary file 3 — Data S3. Morphology‐euclidean distances R code. [file ECE3-6-2886-s003.rtf]

########## traitDistMatrix returns two matrices: 1) is a distance matrix for trait values and the other is the same matrix but scaled by branch lengths - this is similar to rate of trait change per unit branch lengths.traitDistMatrix <- function(traits, phy, mode, p){	d <- treedata(phy, traits, sort=T)	phy <- d$phy	traits <- d$data	n <- nrow(traits)	nc <- ncol(traits)	dist.phy <- cophenetic(phy)	if(nc==1){		dist.morph <- matrix(NA, nrow=n, ncol=n)		for(i in 1:n){			for(j in 1:n){				dist.morph[i,j] <- dist(traits[c(i,j)], method=mode, p=p)			}		}	}	else{		dist.morph <- matrix(NA, nrow=n, ncol=n)		for(i in 1:n){			for(j in 1:n){				dist.morph[i,j] <- dist(traits[c(i,j),])			}		}	}	dist.morph.phy <- dist.morph	for(i in 1:n){		for(j in 1:n){			if(i==j){				dist.morph.phy[i,j] <- 0			}			else{				dist.morph.phy[i,j] <- dist.morph[i,j]/dist.phy[i,j]			}		}	}	dimnames(dist.morph) <- dimnames(dist.morph.phy) <- dimnames(dist.phy)	return(list(dist.morph=dist.morph, dist.phy=dist.phy, dist.morph.phy=dist.morph.phy))}################## Hellinger returns Hellinger distances and inner terms for a pair of niche models.  Assumes models have already been normalized to sum to 1.  Stack is a raster stack object and i and j index the models for which to compute distances.Hellinger <- function(stack,i,j){	X <- stack[[i]]	Y <- stack[[j]]	ex <- extent(X)	ey <- extent(Y)		minx <- max(c(ey@xmin, ex@xmin))	maxx <- min(c(ey@xmax, ex@xmax))	miny <- max(c(ey@ymin, ex@ymin))	maxy <- min(c(ey@ymax, ex@ymax))	ext <- extent(c(minx,maxx,miny,maxy))	X <- crop(X,ext)	Y <- crop(Y, ext)		x <- getValues(X)	y <- getValues(Y)	coords <- xyFromCell(X, 1:ncell(X))	xy <- cbind(coords, x,y)	nas <- which(is.na(xy[,3]))	if(length(nas)>0){xy <- xy[-nas,]}	nas <- which(is.na(xy[,4]))	if(length(nas)>0){xy <- xy[-nas,]}	x <- xy[,3]/sum(xy[,3])	y <- xy[,4]/sum(xy[,4])	inner <- sqrt(x)-sqrt(y)	H <- sqrt(sum(inner^2))	inner <- cbind(xy[,1:2], inner, inner^2)	colnames(inner)[4] <- "inner^2"	return(list(H=H, inner=inner))}##example usagerequire(raster)require(ape)require(vegan)require(geiger)ALL <- stack("niche_model_files")names(ALL) <- "species"PHY <- read.tree("tree_file")X <- read.csv("trait_file")Hdist <- matrix(0, nrow=length(files), ncol=length(files))rownames(Hdist) <- colnames(Hdist) <- speciesfor(i in 1:(nrow(Hdist)-1)){	for(j in (i+1):nrow(Hdist)){		H <- Hellinger(ALL, i, j)		Hdist[j,i] <- H$H		Hdist[i,j] <- H$H		#write.csv(H$inner, paste(dir, species[i], "_vs_", species[j],"_Hellinger_comparisons_", date, ".csv", sep=""))		print(round(Hdist,4))	}}distances <- traitDistMatrix(X, PHY, mode="euchlidean", p=2)morphHellinger <- mantel(Hdist, distances$dist.morph, permutations=999)mantelSumm <- cbind(morphHellinger$statistic, morphHellinger$signif)colnames(mantelSumm) <- c("r", "Pvalue")
